# Response to starvation and microbial community composition in microbial fuel cells enriched on different electron donors

**DOI:** 10.1111/1751-7915.13449

**Published:** 2019-06-22

**Authors:** Soroush Saheb‐Alam, Frank Persson, Britt‐Marie Wilén, Malte Hermansson, Oskar Modin

**Affiliations:** ^1^ Department of Architecture and Civil Engineering Division of Water Environment Technology Chalmers University of Technology Gothenburg Sweden; ^2^ Chemistry and Molecular Biology University of Gothenburg Gothenburg Sweden

## Abstract

In microbial fuel cells (MFCs), microorganisms generate electrical current by oxidizing organic compounds. MFCs operated with different electron donors harbour different microbial communities, and it is unknown how that affects their response to starvation. We analysed the microbial communities in acetate‐ and glucose‐fed MFCs and compared their responses to 10 days starvation periods. Each starvation period resulted in a 4.2 ± 1.4% reduction in electrical current in the acetate‐fed MFCs and a 10.8 ± 3.9% reduction in the glucose‐fed MFCs. When feed was resumed, the acetate‐fed MFCs recovered immediately, whereas the glucose‐fed MFCs required 1 day to recover. The acetate‐fed bioanodes were dominated by *Desulfuromonas* spp. converting acetate into electrical current. The glucose‐fed bioanodes were dominated by *Trichococcus* sp., functioning as a fermenter, and a member of *Desulfuromonadales*, using the fermentation products to generate electrical current. Suspended biomass and biofilm growing on non‐conductive regions within the MFCs had different community composition than the bioanodes. However, null models showed that homogenizing dispersal of microorganisms within the MFCs affected the community composition, and in the glucose‐fed MFCs, the *Trichococcus* sp. was abundant in all locations. The different responses to starvation can be explained by the more complex pathway requiring microbial interactions to convert glucose into electrical current.

## Introduction

Microbial electrochemical technologies, such as the microbial fuel cell (MFC), have been studied extensively during the last two decades (Santoro *et al*., 2017). In an MFC, electroactive microorganisms oxidize organic compounds and deliver electrons to the anode. The electrons travel through an external circuit to the cathode where a reduction reaction takes place. If an electron acceptor with high reduction potential such as oxygen is present, the overall redox reaction is thermodynamically favourable and electrical energy can be harvested from the system (Modin and Gustavsson, 2014). One potential application of MFCs is electrical current production from wastewater (Liu *et al*., 2004). However, there is a range of derivative technologies, which may have greater promise for practical application (Modin and Aulenta, 2017).

A wide variety of electron donors ranging from simple molecules, such as organic acids, to complex mixtures of compounds, such as those present in wastewater, can be used for electrical current generation in MFCs (Pandey *et al*., 2016). The electron donor affects the magnitude of the generated electrical current (Liu *et al*., 2009) as well as the composition of the microbial community catalysing the electrode reactions (Koch *et al*., 2018). Acetate and glucose are examples of two different electron donors, which have been used in several MFC studies. Acetate is non‐fermentable and can be oxidized directly into electrical current by *Geobacter sulfurreducens* (Bond and Lovley, 2003). Although acetate‐fed mixed‐culture bioanodes can be quite diverse, they are often dominated by *Geobacter* spp. (Yates *et al*., 2012; Lewis *et al*., 2018). Glucose conversion at bioanodes can occur through different pathways. *Rhodoferax ferrireducens* (Chaudhuri and Lovley, 2003), *Klebsiella pneumoniae* (Zhang *et al*., 2008) and an *Aeromonas* sp. (Chung and Okabe, 2009) were shown to directly convert glucose into electrical current. However, several studies have suggested that in mixed‐culture systems, electroactive bacteria often coexist with fermentative bacteria and use fermentation products such as acetate and hydrogen for generation of electrical current (Freguia *et al*., 2008; Chae *et al*., 2009). For example, one study found *Geobacter* spp. in association with *Clostridium* spp. (Xing *et al*., 2009). In this case, the *Clostridium* spp. likely performed fermentation of glucose and generated acetate and other products, which could be utilized by the electroactive *Geobacter* spp. Such associations between electroactive bacteria and fermenters have also been observed with other fermentable electron donors (Parameswaran *et al*., 2009).

It is clear that bioanodes enriched on different electron donors develop different microbial communities and this affects the performance of MFCs (Zhang *et al*., 2011). The highest electrical current densities have typically been obtained with acetate‐fed bioanodes (e.g. Chen *et al*., 2012). However, the differences between bioanodes enriched on different electron donors with respect to other performance parameters, such as the response to starvation, have not been investigated. Starvation is a disturbance that is relevant in all environmental biotechnologies as systems sometimes must be inactive because of technical problems or maintenance. Starvation can lead to death, spore formation or a state of extremely slow growth in bacteria (Gray *et al*., 2019). Understanding the effects of starvation is particularly relevant when MFCs are used as sensors for biochemical oxygen demand since the electrochemical activity of the bioanodes must be maintained even if the sensors are exposed to water with low concentration of electron donor. Previously, it was shown that acetate‐fed bioanodes can retain some bioelectrochemical activity when stored in a refrigerator at 4°C for 5 weeks (Saheb Alam *et al*., 2015). Another study showed that acetate‐fed bioanodes could survive a starvation period for 5 days when operated in open‐circuit mode while closed‐circuit conditions improved the resilience up to 11 days (Ruiz *et al*., 2015). To our knowledge, there is no previous study comparing the responses to starvation by parallel MFCs operated under identical conditions but fed with different electron donors and having different microbial community compositions.

Another aspect that is only rarely studied in MFCs is the composition of biomass growing in non‐conductive regions. For example, biofilm growing near the gas‐diffusion cathode may scavenge oxygen and organic electron donor. Cells of non‐electrochemically active microorganisms may detach from this biofilm and attach on the bioanode, affecting its microbial community composition. Microorganisms growing in the bulk liquid may also attach on the bioanode. To our knowledge, there is no study that systematically quantifies how suspended‐, and non‐electroactive microorganisms affect the community assembly of bioanodes in MFCs fed with different electron donors.

The goal of this study was to compare the response to starvation by MFCs enriched on acetate and glucose. We hypothesized that the bioanodes in the two types of MFCs would have different microbial community composition and convert the respective electron donors to electrical current through different pathways, and that this would lead to differences in how they responded to periods of starvation. To get a complete picture of the microbial communities in the MFCs, we sampled bioanodes as well as biomass suspended in the liquid and biomass growing on non‐conductive surfaces near the gas‐diffusion cathodes. Using null models, we quantified the extent to which random dispersal of biomass from non‐conductive locations in the MFCs affected the bioanode community.

## Results

Eight single‐chamber MFCs were connected two‐and‐two in four hydraulic loops (Figs S1 and S2). Four of the MFCs (named MFC0, MFC1, MFC2 and MFC3) were fed with acetate and four (named MFC4, MFC5, MFC6 and MFC7) were fed with glucose. The MFCs were exposed to three 10 days starvation periods.

### Steady‐state performance of the microbial fuel cells

After a start‐up period of 20 days, the MFCs were deemed to have reached steady‐state performance (Fig. S3). With an external resistance of 100 ohm, the peak electrical current density was 1.18 ± 0.04 A m^−2^ in the acetate‐fed MFCs and 0.93 ± 0.03 A m^−2^ in the glucose‐fed MFCs (means and standard deviations calculated for day 20–24). This corresponded to power densities of 176 ± 11 mW m^−2^ (2767 ± 11 mW m^−3^) for the acetate‐fed MFCs and 109 ± 7 mW m^−2^ (1707 ± 108 mW m^−3^) for the glucose‐fed MFCs. The coulombic efficiencies were 29–47% in the acetate‐fed MFCs and 22–28% in the glucose‐fed MFCs. In general, the four MFCs fed with the same electron donor showed highly reproducible electrical current densities. The variation between individual MFCs was 1–8% among acetate‐fed MFCs and 0–6% among glucose‐fed MFCs.

The power output generated by an MFC is maximized when the external resistance equals the internal resistance (Logan *et al*., 2006). Thus, the power output generated by acetate‐ and glucose‐fed MFCs for a given resistance of 100 ohm, as shown above, does not allow comparison of the optimal performance of the two types of MFCs. To get more information about the maximum power output and internal resistance of the MFCs, polarization tests were carried out on days 15, 66 and 98 **(**Fig. S4). In the acetate‐fed MFCs, the internal resistance measured as the slope of the linear region of the polarization curves was quite constant throughout the run going from 133 ± 2 ohm (0.166 ± 0.002 ohm m^−2^) on day 15 to 128 ± 3 ohm (0.161 ± 0.004 ohm m^−2^) on day 98. The maximum power output was the highest on day 15 (200 ± 16 mW m^−2^) and lower on days 66 and 98 (156 ± 9 and 160 ± 6 mW m^−2^). In the glucose‐fed MFCs, the internal resistance increased from 245 ± 62 ohm (0.307 ± 0.077 ohm m^−2^) on day 15 to 321 ± 24 ohm (0.403 ± 0.030 ohm m^−2^) on day 98. At the same time, the maximum power output went from 109 ± 20 to 70 ± 5 mW m^−2^. The polarization curves show a deteriorating performance of the MFCs over time, particularly in the glucose‐fed MFCs. A power overshoot phenomenon was observed on day 15 for the glucose‐fed MFCs. Power overshoots are frequently observed in MFC studies and have been associated with the anode performance in air‐cathode MFCs (Winfield *et al*., 2011). The phenomenon could be caused by depletions of ions and electron donors in the anolyte near the anode at high current densities (Ieropoulos *et al*., 2010). The biofilm maturity (Winfield *et al*., 2011) and the resistor value and anode potential at which it was acclimated (Hong *et al*., 2011) are also important factors. In this study, the MFCs were started‐up at a high resistance of 1000 ohm. When the resistance value was decreased to lower values during the polarization test, the young anode‐attached biofilm was likely unable to oxidize electron donor and transfer electrons at a higher rate, leading to power overshoot. In the later polarization test, the MFCs were adapted to 100 ohm resistances and the power overshoot phenomenon was much less severe.

### Response to starvation

**Figure 1:**
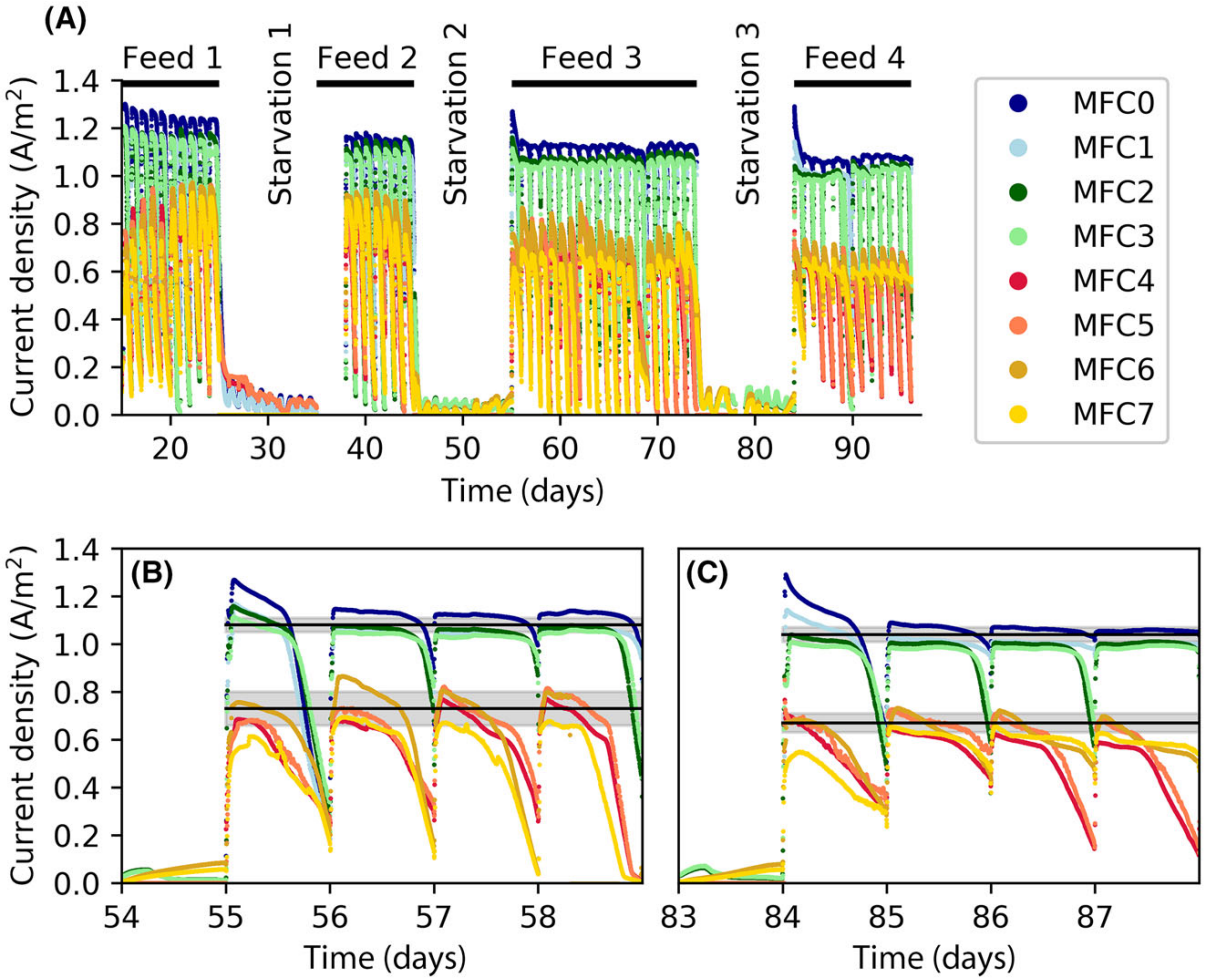
A. Electrical current density generated in the microbial fuel cells (MFCs) during the feed and starvation periods. B. Zoom in on the period right after starvation period 2. C. Zoom in on the period right after starvation period 3. The grey regions in B–C are provided as references to show the average±standard deviation of the peak electrical current densities generated in the respective feed period. The MFCs were fed once per day.

The electrical current generation before, during and after the three starvation periods is shown in Fig. 1A. After each starvation period, the electrical current quickly recovered to a stable value. In the acetate‐fed MFCs, the recovery was immediate, whereas the glucose‐fed MFCs required about 1 day to return to a stable value (Fig. 1B and C).

During each starvation period, half of the MFCs were operated with open circuit and the other half with closed circuit (100‐ohm). With closed circuit during starvation, a small increase in electrical current was observed directly after fresh medium solution was added, even though it did not contain electron donor (Fig. 1A). The maximum electrical current density recorded during the starvation phase, 0.1–0.2 A m^−2^, was 7–13% of the value produced during normal operation. The acetate‐fed MFC operated with open circuit during starvation generated a high current peak after electron donor feed was resumed and the circuit was closed (see MFC0‐1). This was not observed for the glucose‐fed MFCs (Fig. 1B and C).

**Figure 2:**
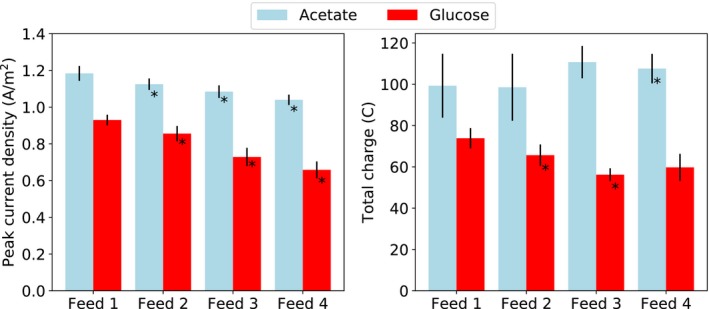
Average and standard deviations of the peak electrical current density or the total charge passed by the microbial fuel cells during a day in the feed periods. Asterisk (*) marks that there was a statistically significant difference in comparison with the previous feed period (*P* < 0.05, *n* = 4, paired sample *t*‐test).

Figure 2 shows the peak electrical current density and daily charge generation during each feed period (the first day just after starvation was excluded from the calculation). Each starvation period resulted in a significant reduction in the peak electrical current density in the following feed period. However, there was no clear effect on the charge generation. In the acetate‐fed MFCs, a starvation period resulted in a 4.2 ± 1.4% drop in the electrical current density. In the glucose‐fed MFCs, the drop was 10.8 ± 3.9%, which was significantly higher than in the acetate‐fed MFCs (*P* < 0.001, Welch's ANOVA). In the acetate‐fed MFCs, a slight difference was observed between those operated with open circuit, which had a reduction of 3.1 ± 0.7% and those operated with closed circuit, which had a reduction of 5.3 ± 1.0% (*P* < 0.01, Welch's ANOVA). No such difference was seen for the glucose‐fed MFCs.

### Ability to use other electron donors

The ability of the MFCs to generate electrical current from alternative electron donors was investigated in two of the MFCs at the end of the experiment. Formate feed resulted in the highest electrical current for both acetate‐ and glucose‐enriched MFCs. Switching from acetate to other electron donors in MFC1 led to lower electrical current and charge. On the other hand, replacing glucose in MFC5 with any of the alternative electron donors resulted in higher electrical current and charge. When the acetate‐enriched MFC was fed with glucose, the average charge was 42% lower than when fed with acetate. However, the glucose‐enriched MFC could produce 58% more charge when it was fed with acetate (Fig. S5). The shape of the electrical current versus time curve during a batch run also looked different for the different electron donors (Fig. S6). With formate, both MFC1 and MFC5 generated a sharp current peak directly after feeding. MFC5 also responded rapidly to acetate, lactate and propionate. The peaks in MFC1 were more drawn out and appeared later when it was fed with glucose, lactate, propionate and butyrate.

The ability of the microbial communities growing on the electrodes to use the electrode itself as electron donor was investigated in two MFCs at the end of the experiment. Cyclic voltammetry showed that the glucose‐enriched electrode in MFC7 produced higher cathodic current than the acetate‐enriched electrode in MFC3 (Fig. S7). During prolonged enrichment at first −0.65 V versus SHE and later −0.8 V versus SHE with hydrogen ions and carbon dioxide as the possible electron acceptors, the glucose‐enriched electrode initially produced higher cathodic current than the acetate‐enriched electrode, but after about 2 months of operation, both converged to a current density of about 0.1 A m^−2^ (Fig. S8).

### Microbial community composition

**Figure 3:**
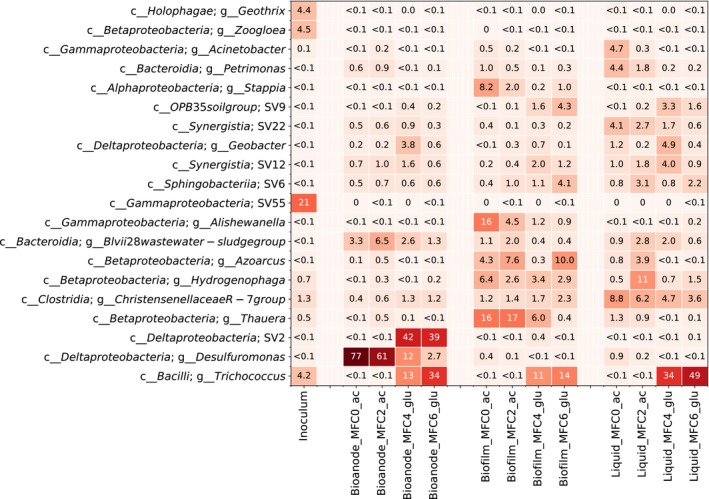
Heatmap showing the percentage relative abundance of the most abundant genera. On the vertical axis, the abbreviation *g*__ refers to genus and *c*__ refers to class. Unclassified sequence variants (SVs) are noted as SV and a number. The labels on the horizontal axis show the sample type, the microbial fuel cells and the type of electron donor used as feed (*ac* = acetate and *glu* = glucose).

Biomass samples for microbial community analysis were collected from four of the MFCs, one from each hydraulic loop. In each MFC, biomass was sampled from the bioanode, the liquid suspension and the partially aerobic biofilm growing near the gas‐diffusion cathode. A heatmap with the most abundant genera is shown in Fig. 3. A categorization based on class is shown in Fig. S9. For the bioanodes, *Deltaproteobacteria* was a major class representing 45–79% of the reads. In the acetate‐fed MFCs, a single genus, *Desulfuromonas*, was dominating with 61–77% of the reads. This genus had much lower abundance in the glucose‐fed MFCs, 12% in MFC4 and 2.7% in MFC6. In the glucose‐fed MFCs, a different taxon, unclassified at the genus level but belonging to the *Desulfuromonadales* order, was more abundant (39–42%). The glucose‐fed bioanodes also contained a large fraction of *Trichococcus* sp. (13–34%).

The microorganisms in the biofilms were distributed between several different classes including *Alpha‐*,* Beta*‐ and *Gammaproteobacteria* as well as *Bacteroidia*. *Bacilli*, which includes *Trichococcus* sp., was mainly present in the glucose‐fed biofilms (11–15%) and nearly absent in those fed with acetate (< 0.1%). The genera *Thauera*,* Azoarcus, Hydrogenophaga, Alishewanella* and *Stappia* were also abundant in the biofilms enriched both on acetate and glucose. The liquid samples varied between individual MFCs. *Trichococcus* sp. was dominating in the liquid from the glucose‐fed MFCs. *Clostridia*, particularly a taxon classified as *Christensenellaceae R‐7 group*, were more abundant in the liquid samples than in the bioanode or biofilm samples in both types of MFCs. Archaea had low abundance in the MFCs. In the liquid of the glucose‐fed MFCs, *Methanomicrobia* made up 2.3–3.0% of the reads. The rest of the samples had < 0.7% Archaea.

### Differences in community composition

Two dissimilarity indices were used to analyse differences in community composition between samples. The incidence‐based index, 1‐C_0_, considers only the presence/absence of sequence variants (SV) in the samples. The index 1‐C_1_ gives weight to each SV according to its relative abundance.

**Figure 4:**
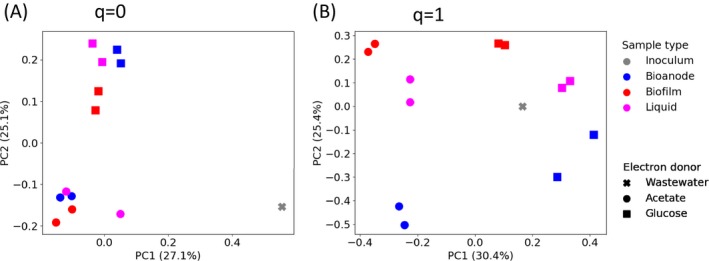
Principal coordinate analysis based on pairwise dissimilarity matrices calculated using (A) 1‐C_0_ (presence/absence data) or (B) 1‐C_1_ (the relative abundance of sequence variants is taken into account).

The differences in community composition between samples were illustrated using ordination (Fig. 4). Considering only the presence/absence of SVs (1‐C_0_), the samples clustered based on electron donor. When the relative abundance of SVs was taken into account (1‐C_1_), the samples clustered more according to type, i.e. acetate‐fed bioanodes formed one cluster and acetate‐fed biofilm formed another.

**Figure 5:**
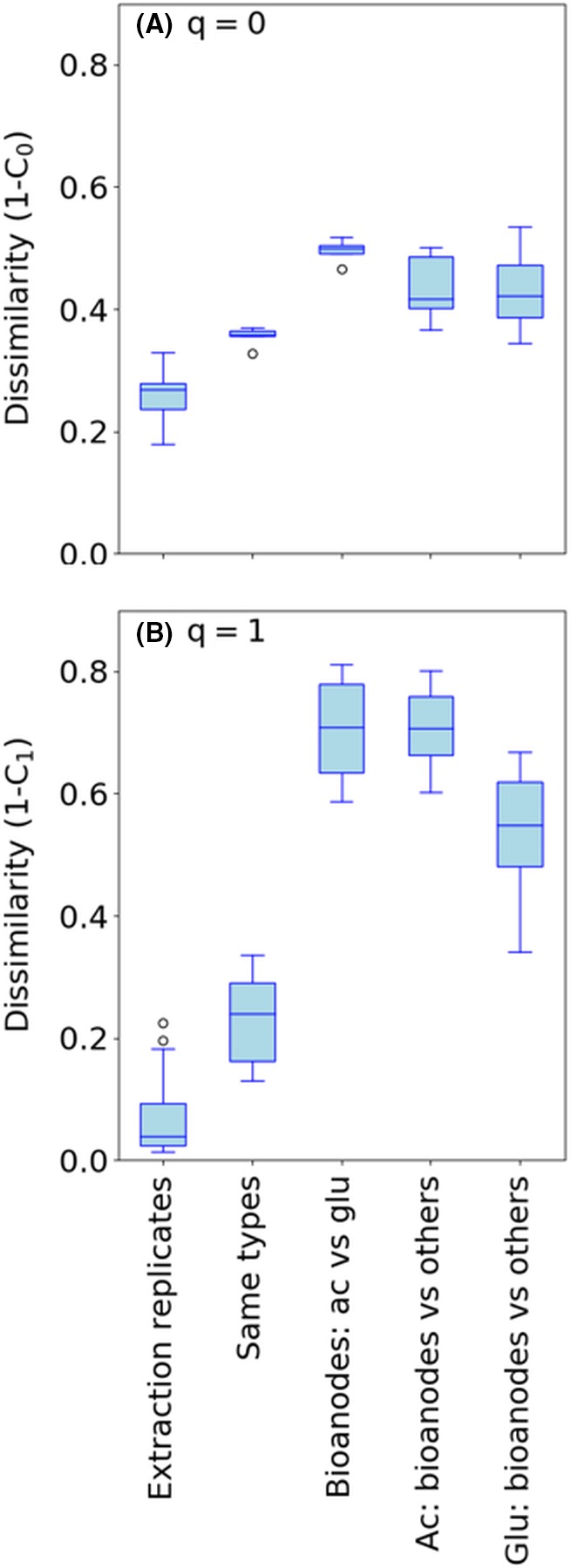
Dissimilarity values for different categories of samples calculated using (A) 1‐C_0_ (presence/absence data) or (B) 1‐C_1_ (the relative abundance of sequence variants is taken into account). *Same types* refer to samples of the same type (e.g. bioanodes), fed with the same electron donor (e.g. acetate), but collected from different MFCs (e.g. MFC0 and MFC2). *Ac* refers to acetate, and *glu* refers to glucose.

The average pairwise dissimilarities between different categories of samples are shown in Fig. 5. The values for extraction replicates show that the variation between samples caused by methodological issues (DNA extraction, PCR, sequencing) affected mostly rare SVs since 1‐C_0_ (0.26 ± 0.03) was much higher than 1‐C_1_ (0.06 ± 0.05). The values for samples of the same type show differences between the parallel reactors. In comparison with dissimilarity values caused by methodological issues and parallel reactors, the dissimilarities between bioanodes from MFCs fed with acetate and glucose were significantly higher (*P* < 0.001, Welch's ANOVA). The dissimilarities between bioanodes and other types of biomass (i.e. biofilm and liquid) in the MFCs fed with the same electron donor were also significantly higher than the dissimilarity caused by methodological issues (*P* < 0.05, Welch's ANOVA). Moreover, the bioanodes and other biomass were more dissimilar to each other in the acetate‐fed MFCs compared to the glucose‐fed MFC when considering the relative abundance of SVs (1‐C_1_) (*P* < 0.01, Welch's ANOVA).

The observed dissimilarity between samples of different types could have been caused both by differences in community composition and by differences in richness (i.e. the number of SVs found in different samples) (Chase *et al*., 2011). To distinguish between these two effects, null models were used. The observed dissimilarities between the bioanode, the liquid and the biofilm samples in each MFC were compared to a null distribution of dissimilarity values. The null distribution was based on communities randomly assembled from a regional pool of SVs. For the acetate‐fed MFCs, this regional pool consisted of all the SVs detected in all the samples from the acetate‐fed MFCs. Similarly, for the glucose‐fed MFCs, the regional pool consisted of all the SVs detected in the samples from the glucose‐fed MFCs. The RC_0_ (incidence‐based) and RC_1_ (relative abundance‐based) indices, constrained between −1.00 and + 1.00 quantify whether two sampled community are less (−1.00) or more (+1.00) dissimilar to each than what would be expected is the communities, were randomly assembled from the regional species pool. When comparing the bioanodes to the liquid and biofilm samples, the observed incidence‐based dissimilarity index (1‐C_0_) was lower than the null expectation (RC_0_ = −1.00) for the acetate‐fed MFCs, and lower or similar to the null expectation for the glucose‐fed MFC (RC_0_ was −1.00 to + 0.94). The observed relative abundance‐based dissimilarity index (1‐C_1_) was higher than the null expectation for acetate‐fed MFCs (RC_1_ = +1.00) and higher or similar to the null expectation for the glucose‐fed MFCs (RC_1_ was + 0.59 to + 1.00) (Table S1 and S2). These results show that when we only consider detection of SVs, the bioanode, liquid and biofilm samples had very similar community composition. RC_0_ values close to −1.00 suggest that there is a homogenizing dispersal of cells within the MFCs. However, when we consider the relative abundance of SVs, the community composition of samples of different types is very different from each other. This suggests a deterministic selection of taxa depending on the location within the MFC. The bioanode, liquid, and non‐conductive surface exert different selection pressures and allow different taxa to grow. The null model results are more variable for the glucose‐fed MFCs with several intermediate RC_0_ and RC_1_ values. This suggests that microbial community assembly in the glucose‐fed MFCs was more similar to a random process than in the acetate‐fed MFCs.

## Discussion

### Differences in microbial community composition and electron donor oxidation pathways

Both the acetate‐ and glucose‐fed bioanodes were dominated by *Deltaproteobacteria*, but by different members of the class. The acetate‐fed bioanodes were dominated by *Desulfuromonas* spp. This taxon has previously been observed on bioanodes generating current from ethanol (Kim *et al*., 2007), swine and bovine wastewater (Rago *et al*., 2018) and seawater sediments (Bond *et al*., 2002). Tender *et al*. (2002) found microorganisms related to *Desulfuromonas acetoxidans* associated with the anode in a sediment MFC. A pure culture of this species could generate current from acetate (Bond *et al*., 2002).

In the glucose‐fed bioanodes, a bacterium of the *Desulfuromonadales* order, unclassified at the genus level, was highly abundant. Members of *Desulfuromonadales*, which include both *Desulfuromonas* spp. and *Geobacter* spp., are known to be capable of extracellular electron transfer (Poddar and Khurana, 2011; Ishii *et al*., 2013; Greene, 2014). A *Trichococcus* sp. was also highly abundant on the bioanodes as well as in other locations of the glucose‐fed MFCs. *Trichococcus* spp. are aerotolerant fermenters known to produce acetate and lactate from glucose (Rainey, 2015). This microorganism likely fermented glucose, and the fermentation products served as electron donors for the electroactive *Desulfuromonadales* sp. This pathway for glucose oxidation into electrical current is also supported by the tests with alternative electron donors, in which the glucose‐enriched bioanode produced higher electrical current densities when it was fed with VFAs than when it was fed with glucose. The results are also in line with previous studies showing that glucose oxidation in mixed‐culture bioanodes is typically accomplished by an association of fermentative and electrogenic bacteria (Freguia *et al*., 2008). For example, *Geobacter* sp. has been found in combination with fermentative bacteria (Chae *et al*., 2009; Xing *et al*., 2009) such as *Clostridium* and *Bacilli* (Xing *et al*., 2009; Zhang *et al*., 2011). *Trichococcus* sp. was previously observed in an MFC fed with cattle dung (Zhao *et al*., 2012). However, to our knowledge, the specific association between *Trichococcus* and *Desulfuromonadales* for oxidation of glucose at the bioanode of an MFC has not been reported before. Both VFAs and hydrogen may have served as electron shuttles between *Trichococcus* and *Desulfuromonadales*. Cyclic voltammetry and operation of the electrodes as cathodes showed that the glucose‐enriched electrodes produced higher cathodic current than those enriched on acetate (Figs S7 and S8). Microorganisms containing hydrogenases are known to catalyse cathodic reactions (Rozendal *et al*., 2008; Saheb Alam *et al*., 2018). *Trichococcus collinsii* has previously been shown to produce hydrogen during fermentation (Dębowski *et al*., 2014). Thus, it is likely that the *Trichococcus* sp. observed on the glucose‐fed bioanodes in this study generated both hydrogen and VFAs when fermenting glucose and that the presence of hydrogenases on the bioanode led to catalysis of the hydrogen evolution reaction when the potential of the electrode was lowered.

In both acetate‐ and glucose‐fed MFCs, *vadinBC27* and *Blvii28* – two potentially electroactive genera – were more abundant in the bioanode samples in comparison with other sampling locations within the MFCs. The genus *vadinBC27* and related genera within *Bacteroidetes* have previously been observed on bioanodes in MFCs fed with swine manure (Rago *et al*., 2017). Interestingly, *Geobacter* spp., which have been found to dominate in many other studies with both acetate‐ and glucose‐fed bioanodes (Torres *et al*., 2009; Kiely *et al*., 2011; Yates *et al*., 2012; Lewis *et al*., 2018), had low relative abundance in the MFCs. However, both *Geobacter* spp. and *Desulfuromonas* spp. are known as dissimilatory Fe(III) reducers (Lonergan *et al*., 1996), and the ability to use an anode as electron acceptors appears to be spread within this group. The specific taxon that develops dominance in an MFC may depend on the inoculum, the experimental conditions and stochasticity during community development.

Abundant taxa in the biofilm and liquid of the MFCs likely functioned as oxygen scavengers or fermenters. For example, *Thauera* spp. and *Azoarcus* spp. are known to take up acetate under aerobic conditions (Thomsen *et al*., 2007). *Alishewanella* spp. and *Stappia* spp. have been found in several different environments (Weber and King, 2006; Jung and Park, 2013) and probably had the same role. *Hydrogenophaga* spp. are known to be aerobic and capable of utilizing several organic compounds (Yoon *et al*., 2008). In the glucose‐fed MFCs, *Trichococcus* was the most abundant taxon both in the liquid and in the biofilms and was likely mainly responsible for fermentation of glucose. *Christensenellaceae* spp. are anaerobic and could also have contributed to fermentation of glucose (Morotomi *et al*., 2012). In the acetate‐fed MFCs, they may have fermented extracellular polymeric substances, decay products and metabolites from other microorganisms. The relative abundance of Archaea was low in the MFCs. The reason was probably temporary exposure to oxygen. In the end of each feed cycle, the organic electron donor was typically depleted in the system and oxygen may have entered through the gas‐diffusion cathode, limiting the growth of methanogens.

The microbial communities that developed on acetate‐ and glucose‐fed bioanodes were clearly different. It is also notable that the dissimilarity index between the bioanodes fed with different electron donors was higher when we consider ‘common’ SVs (1‐C_1_) than when we consider presence/absence data (1‐C_0_) (Fig. 5) suggesting that electron donor had a large effect on shaping the communities.

Dispersal of microorganisms is an important factor for the structure and composition of microbial communities in nature and in bioreactors (Sloan *et al*., 2006; Ofiteru *et al*., 2010). In MFCs, bioanode communities may well be affected by immigration from biomass growing in other locations of the MFCs (e.g. the biofilms and the liquid); however, this has not been addressed previously. When we investigated differences in community composition using a presence/absence‐based dissimilarity index (1‐C_0_) and a null model, we see that the microbial community composition in samples collected from different locations within the MFCs is more similar to each other than expected by chance (RC_0_ = −1.00). This suggests homogenizing dispersal of microorganisms within the MFCs. For example, microorganisms detached from the biofilms attach on the bioanodes and vice versa. However, when we consider the relative abundance of SVs (1‐C_1_), we see large differences in community composition (RC_1_ = +1.00). This shows that even if microbial cells are dispersed within the MFC, there is a strong selection due to the local environment, i.e. the location where the microorganisms can grow is very specific. A previous study of an acetate‐fed single‐chamber MFCs also found a large difference in community composition between the bioanode and the biofilm attached on the gas‐diffusion cathode (Daghio *et al*., 2015). However, that study only focused on the most abundant taxa. In our study, the dissimilarity in community composition between sample types was smaller in the glucose‐fed MFCs than in the acetate‐fed MFCs. The dissimilarity between the bioanodes and the liquid samples in the glucose‐fed MFCs could even be explained by random assembly. The reason for this is that glucose is a fermentable electron donor, and fermentative microorganisms are not dependent on access to an electron donor such as the anode or oxygen diffusing in through the cathode and can therefore grow in all locations of the MFC. Indeed, *Trichoccocus* sp. was found everywhere in the glucose‐fed MFCs (Fig. 3).

### Differences in the response to starvation

The acetate‐ and glucose‐fed MFCs had different responses to 10 days starvation periods. The acetate‐fed MFCs had a smaller drop in peak current density over each starvation period in comparison with the glucose‐fed MFCs (4.2 ± 1.4% versus 10.8 ± 3.9%). No previous studies report percentage reductions in the steady‐state electrical current generation after starvation periods. However, some studies have focused on the first batch cycles after electron donor feed is resumed. Kaur *et al*. (2014) investigated starvation as a method to inhibit methanogens in an acetate‐fed MFC. A 12 day starvation resulted in a reduction in coulombic efficiency in the first batch cycle after acetate feed was resumed. Current densities were not reported. Gao *et al*. (2014) reported that MFCs could handle 7 day periods of starvations; however, only spikes of acetate were provided in that study, and changes in the steady‐state current generation were not reported. The most extensive previous study on the effects of starvation was carried out by Ruiz *et al*. (2015). They observed that an acetate‐fed MFC operated in closed‐circuit mode during starvation could handle 11 days without a significant decrease in current density. However, for an MFC operated in open‐circuit mode during starvation, 7 days resulted in a 25% drop in current density once acetate feed was resumed, although the original performance could be recovered in subsequent feed cycles. Irreversible reduction in current density was observed after 21 days of starvation (Ruiz *et al*., 2015). In our study, we did not see any clear difference between open‐circuit and closed‐circuit conditions. Differences between the studies may be explained by differences in MFC performance. In the study by Ruiz *et al*. (2015), the MFCs tested in closed‐circuit and open‐circuit starvation conditions generated about 0.0027 and 0.008 A m^−2^, respectively, whereas the acetate‐fed MFCs in our study generated about 1.18 ± 0.04 A m^−2^. In bioanodes generating low current density, the respiration rate of the electroactive microorganisms may be much lower than their maximum capacity. Thus, if a certain fraction of the microorganisms dies during a starvation period, the remaining part of the biofilm may still be able to make up for the loss by respiring at a higher rate. On the other hand, in an electroactive biofilm where the microorganisms are respiring at a rate near their maximum, a reduction in the electroactive biofilm will lead to a reduction in the generated current density, which was observed as a 4.2 ± 1.4% reduction in our study.

For glucose‐fed MFC, there are fewer starvation studies. Chang *et al*. (2004) studied the effects of starvation on an MFC fed with a glucose/glutamic acid solution. A starvation period of 11.25 days resulted in a recovery period of 3.33 days before the electrical current reached similar values to before starvation. The recovery period can be compared to the 1 day required for our glucose‐fed MFC to reach a stable peak current after a starvation period. A difference to our study is that Chang *et al*. (2004) operated their system with continuous feed and they did not report any irreversible reduction in electrical current density, whereas we had a semi‐continuous feed and observed a 10.8 ± 3.9% reduction after each starvation period.

A reason for the reduction in electrical current density could be that each starvation led to some decay of the electroactive biomass on the bioanodes. If the dead biomass remained on the anode, it could have prevented new bacteria from colonizing the surface, thereby preventing the electrical current from reaching its pre‐starvation value. The more severe effect in the glucose‐fed MFCs was likely caused by the requirement of two participating microorganisms (the fermenter and the electroactive) to oxidize glucose into electrical current. A starvation period could lead to an imbalance in the partnership between the two. For example, spatial links between the two types of microorganisms could be disrupted. Presumably, the most efficient conversion of glucose to electrical current would occur if fermentative bacteria were located right next to electroactive bacteria on the bioanode, leading to locally high concentrations of fermentation products, fast reaction rates and high electrical current generation. If either the fermenter or the electroactive bacteria decayed during the starvation phase, such links would be broken, and the fermentation products would have to diffuse a longer distance before they could be utilized for electrical current generation. The more complex pathway and microbial consortium required to oxidize glucose into electrical current may also explain the 1 day recovery period. An electroactive microbial community capable of direct conversion of glucose to electrical current without fermentation may have a different response to starvation.

Contrary to the peak electrical current density, the charge generated per day did not appear to be negatively affected by starvation periods. In some cases, starvation periods even led to more charge being generated. A reason could be that bacteria competing with the electroactive microbes for electron donor decreased in abundance during the starvation period, which meant that a larger fraction of the supplied electron donor was used for current generation. During the starvation tests, low levels of current generation were observed in the MFCs operated with closed circuit. We speculate that this is because low levels of oxygen in the feed led to partial oxidation of biomass in the reactor, which could have served as electron donors for electroactive microbes. Vitamins in the nutrient medium may also have contributed to the current generation.

In conclusion, distinctly different microbial communities developed on the bioanodes in acetate‐ and glucose‐fed MFC. After 10 days starvation periods, the glucose‐fed MFCs had a longer lag phase and a larger percentage drop in steady‐state current density. This can likely be explained by the comparatively more complex microbial community, involving interactions between fermenters and electroactive bacteria, required for the conversion of glucose into electrical current.

## Experimental procedures

### Microbial fuel cells

The eight single‐chamber air‐cathode MFCs each consisted of a cylindrical Plexiglas compartment with a length of 5 cm and a diameter of 4 cm. The anode was a 4 cm diameter carbon cloth (AvCarb 1071 HCB, Fuelcellearth.com) placed on one side of the cylindrical compartment. The gas‐diffusion cathode catalysed by carbon black was placed on the opposite side (Modin and Fukushi, 2012). A glass‐fibre filter separated the cathode from the bulk liquid (see Appendix S1 for further details).

### Operation

The eight MFCs (numbered from 0 to 7) were placed in four hydraulic loops. Nutrient medium was circulated through each loop at 40 ml min^−1^. The total liquid volume of one loop (including tubing and two MFCs) was 160 ml. The reactors were fed once a day. At that time, the recirculation pump was stopped and 46 ml of nutrient medium was pumped into the system during a time period of 5 min. During feeding, an equivalent volume of liquid was simultaneously wasted from the loop (Fig. S2). Because of non‐ideal plug flow during feeding, approximately 8% of the electron donor in the feed was lost in the effluent during feeding.

The nutrient medium consisted of 0.1 g l^−1^ of KCl, 0.6 g l^−1^ of KH_2_PO_4_, 0.25 g l^−1^ of NH_4_Cl, 3 g l^−1^ of NaHCO_3_, 0.1 g l^−1^ of MgCl and 0.03 g l^−1^ of CaCl. Trace element and vitamin solutions, as described by Marshall *et al*. (2012), were also added. Either acetate or glucose was also added to the nutrient medium at a concentration of 1.28 g l^−1^ chemical oxygen demand (COD). Each hydraulic loop was inoculated with 15 ml of activated sludge collected from a municipal wastewater treatment plant.

After a 15 day start‐up period with an external resistance of 1000 ohm, the MFCs were operated with an external resistance of 100 ohm. This resistance value was chosen as a compromise between obtaining a high electrical current generation and coulombic efficiency, which requires a low resistance, and obtaining a high power output, which requires an external resistance value similar to the internal resistance.

### Starvation tests

Three starvation tests were carried out during the experiment. During a starvation test, organic carbon was excluded from the nutrient medium for 10 consecutive days and the MFCs were operated with either open‐circuit conditions or with a 100 ohm resistor placed between the anode and cathode. The starvation tests were carried out on days 25–34, 45–54 and 74–83.

### Alternative electron donor test

Between days 128 and 147 of the experiment, two MFCs (#1 and #5) were alternatively fed with their normal electron donor and another compound. Formate, lactic acid, propionate, butyrate and glucose/acetate were tested. During this test, only one MFC was placed in each hydraulic loop and the feed concentration was lowered to 0.16 g l^−1^ COD to ensure that all organic carbon would be consumed in the MFC during the 1 day feed cycles.

From day 128, the ability of the bioanodes from two of the MFCs (#3 and #7) to function as biocathodes was tested. The gas‐diffusion cathode was replaced with another cylindrical compartment harbouring a 4 cm long, 3 mm diameter platinum‐coated titanium wire (Magneto Special Anodes Bv, Schiedam, The Netherlands). The two compartments were separated by a glass‐fibre filter, and both were filled with nutrient medium without organic carbon. A silver/silver chloride reference electrode, fabricated as described previously (Modin *et al*., 2017), was placed in the compartment hosting the biological electrode. Nutrient medium was also circulated through this compartment at a flow rate of 40 ml min^−1^. The biological electrode was controlled at a potential of −0.65 V versus SHE using a NEV4 potentiostat (NanoElectra). After 128 days of operation, the potential was lowered to −0.8 V versus SHE. The reactors were operated in biocathode mode for a total of 203 days.

### Microbial community analysis

One MFC from each hydraulic loop was sampled for microbial community analysis on day 112 of the experiment. Samples were collected from the inoculum (the activated sludge used to start‐up the MFCs), the bioanodes, the biofilms and the liquid suspension. DNA extraction was carried out using the FastPrep for soil kit. PCR was carried out with the primer pair 515′F (GTGBCAGCMGCCGCGGTAA) and 806′R (GGACTACHVGGGTWTCTAAT) (Caporaso *et al*., 2011; Hugerth *et al*., 2014). Sequencing of pooled samples was carried out using Illumina MiSeq. To assess methodological effects on sequencing results, two bioanodes and two biofilms were cut into six replicate samples and DNA was extracted and processed independently from each replicate. Further details are provided in Appendix S2. There, the reader can also find information about the methods used to process the sequence reads. In brief, we used the UNOISE algorithm (Edgar, 2016) to denoise quality‐filtered reads and generate sequence variants (SVs). The difference between SVs and operational taxonomic units (OTUs), which are conventionally determined by clustering sequences with > 97% nucleotide similarity, is that SVs are true biological sequences that can differ from each other by as little as one nucleotide, whereas OTUs depend on the fixed clustering threshold (see Callahan *et al*., 2017). The raw sequence reads were deposited in the European Nucleotide Archive (project number: PRJEB26776).

The dissimilarity in microbial community composition between pairwise combinations of samples was calculated using the framework for species diversity based on Hill numbers described by Chao *et al*. (2014). The parameter C_q_ represents the local overlap between two samples and can be interpreted as the fraction of SVs in one sample that is shared with the other sample. Consequently, 1‐C_q_ is the dissimilarity, i.e. the fraction of SVs is *not* shared between two samples. The diversity order q determines the importance placed on the relative abundances of the SVs. At diversity order 0, relative abundance is not considered and 1‐C_0_ thus equals the fraction of all detected SVs in one sample that are not shared with the other sample. At diversity order 1, the SVs are weighted according to their relative abundance and 1‐C_1_ can be interpreted as the fraction of ‘common’ SVs not shared. Null models resulting in the Raup–Crick (RC) index were used to distinguish between differences in community composition to difference in alpha diversity (Raup and Crick, 1979). We used the randomization approach presented by Chase *et al*. (2011) and Stegen *et al*. (2013) and applied it to our dissimilarity indices, 1‐C_0_ and 1‐C_1_. We call the indices RC_0_ and RC_1_ for the incidence‐based and relative abundance‐based cases, respectively. A value close to −1.00 indicates that two samples have more similar community composition than expected by chance, whereas a value close to + 1.00 indicates that the samples are more dissimilar than expected by chance. All calculations were carried out using the software qDiv (Modin, 2018).

### Analytical methods

Organic acids and glucose were measured using high‐pressure liquid chromatography as described previously (Saheb Alam *et al*., 2018). The electrical current generation in the MFCs was determined by logging the voltage across the external resistors every 30 s (USB 2011 data logger, National Instruments). The peak electrical current for each batch cycle refers to the highest average electrical current observed during a 5 min period. The charge generated during a batch cycle was determined by integrating the electrical current with respect to time. Polarization analysis of the MFC was carried out by leaving the MFCs in open circuit for 1 h and then sequentially connecting resistors ranging from 3000 ohm to 10 ohm and measuring the cell potential for a period of 15 min at each resistor value. The polarization tests were carried out approximately 3–6 h after feeding. During this time interval, the current generation by the MFCs was typically stable (Fig. 1). Statistical tests were carried out using the Scipy package in Python 3 (Jones *et al*., 2001).

## Conflict of interest

None declared.

## Supporting information


**Fig. S1.** Schematic of a MFC and photo of the experimental setup.
**Fig. S2.** Schematic of a hydraulic loop containing two microbial fuel cells (MFC).
**Fig. S3.** Electrical current density generated by the eight MFCs during (A) the initial 15 days with 1000 ohm resistors, and (B) day 15–25 with 100 ohm resistors.
**Fig. S4.** Polarization experiments carried out during the MFC experiment.
**Fig. S5.** Peak electrical current density (A) and daily charge (B) generated by MFCs when fed with different substrates. MFC1 was enriched on acetate and MFC5 was enriched on glucose. The average of two sequential batch cycles are shown. The error bars show the minimum and maximum values.
**Fig. S6.** Current density versus time for the experiments with different substrates.
**Fig. S7.** Results from cyclic voltammetry carried out in the end of the MFC experiment. MFC3 had been operated with acetate feed and MFC7 with glucose feed. The potential was scanned between + 0.2 and −0.8 V versus SHE in three cycles at a rate of 1 mV s^−1^. The results from the third cycle are shown. Cathodic current is negative.
**Fig. S8.** Prolonged enrichment of the biological electrodes from MFC3 and MFC7 under anaerobic conditions in a solution containing hydrogen ions and carbon dioxide as the only possible electron acceptors. The electrode potential was first controlled at −0.65 V versus SHE and then at −0.8 V versus SHE. Cathodic current is negative.
**Fig. S9.** Heatmap showing the most abundant taxa categorized based on class. On the vertical axis, the abbreviation *c*__ refers to class and *p*__ refers to phylum. The labels on the horizontal axis show the samples type, the MFC, and the type of electron donor used as feed (*ac* refers to acetate and *glu* refers to glucose‐feed). The numbers in the heatmap show percentage relative abundance in the sample.
**Table S1.** Raup‐Crick dissimilarity values calculated based on 1‐C_0_. A value > 0.95 indicates that the difference in community composition between two samples is significantly higher than could be explained by random assembly. A value < −0.95 indicates that the difference is significantly smaller than could be explained by random assembly (see Chase *et al*., 2011).
**Table S2.** Raup‐Crick dissimilarity values calculated based on 1‐C_1_. A value > 0.95 indicates that the difference in community composition between two samples is significantly higher than could be explained by random assembly. A value < −0.95 indicates that the difference is significantly smaller than could be explained by random assembly (see Chase *et al*., 2011).
**Appendix S1.** Description of the gas‐diffusion cathodes.
**Appendix S2.** Microbial community analysis methods.
