# Supplementary material for: Response to starvation and microbial community composition in microbial fuel cells enriched on different electron donors
Source: Microb Biotechnol. 2019 Jun 22;12(5):962–75. doi: 10.1111/1751-7915.13449 (PMC6680615; doi:10.1111/1751-7915.13449)
Supplement: Supplementary file 1 — Fig. S1. Schematic of a MFC and photo of the experimental setup. Fig. S2. Schematic of a hydraulic loop containing two microbial fuel cells (MFC). Fig. S3. Electrical current density generated by the eight MFCs during (A) the initial 15 days with 1000 ohm resistors, and (B) day 15–25 with 100 ohm resistors. Fig. S4. Polarization experiments carried out during the MFC experiment. Fig. S5. Peak electrical current density (A) and daily charge (B) generated by MFCs when fed with different substrates. MFC1 was enriched on acetate and MFC5 was enriched on glucose. The average of two sequential batch cycles are shown. The error bars show the minimum and maximum values. Fig. S6. Current density versus time for the experiments with different substrates. Fig. S7. Results from cyclic voltammetry carried out in the end of the MFC experiment. MFC3 had been operated with acetate feed and MFC7 with glucose feed. The potential was scanned between + 0.2 and −0.8 V versus SHE in three cycles at a rate of 1 mV s−1. The results from the third cycle are shown. Cathodic current is negative. Fig. S8. Prolonged enrichment of the biological electrodes from MFC3 and MFC7 under anaerobic conditions in a solution containing hydrogen ions and carbon dioxide as the only possible electron acceptors. The electrode potential was first controlled at −0.65 V versus SHE and then at −0.8 V versus SHE. Cathodic current is negative. Fig. S9. Heatmap showing the most abundant taxa categorized based on class. On the vertical axis, the abbreviation c__ refers to class and p__ refers to phylum. The labels on the horizontal axis show the samples type, the MFC, and the type of electron donor used as feed (ac refers to acetate and glu refers to glucose‐feed). The numbers in the heatmap show percentage relative abundance in the sample. Table S1. Raup‐Crick dissimilarity values calculated based on 1‐C0. A value > 0.95 indicates that the difference in community composition between two samples is significantly high [file MBT2-12-962-s001.pdf]

Response to starvation and microbial community composition in microbial fuel cells enriched on different electron donors

Soroush Saheb-Alam[a], Frank Persson[a], Britt-Marie Wilén[a], Malte Hermansson[b], Oskar Modin[a*]
[a]Chalmers University of Technology, Department of Architecture and Civil Engineering, Division of Water Environment Technology, Gothenburg, Sweden
[b]University of Gothenburg, Chemistry and Molecular Biology, Gothenburg, Sweden
*Corresponding author: oskar.modin@chalmers.se, +46 31 772 2138

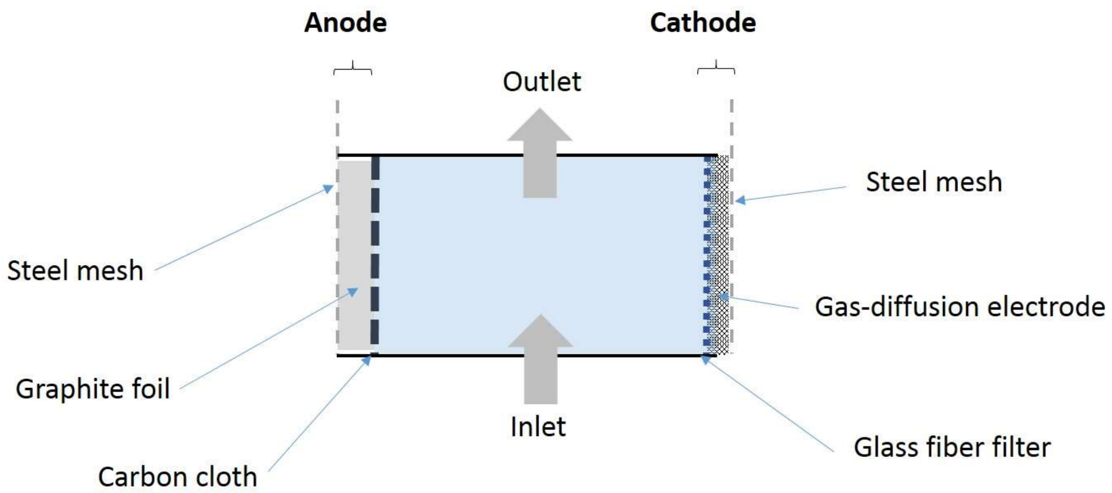

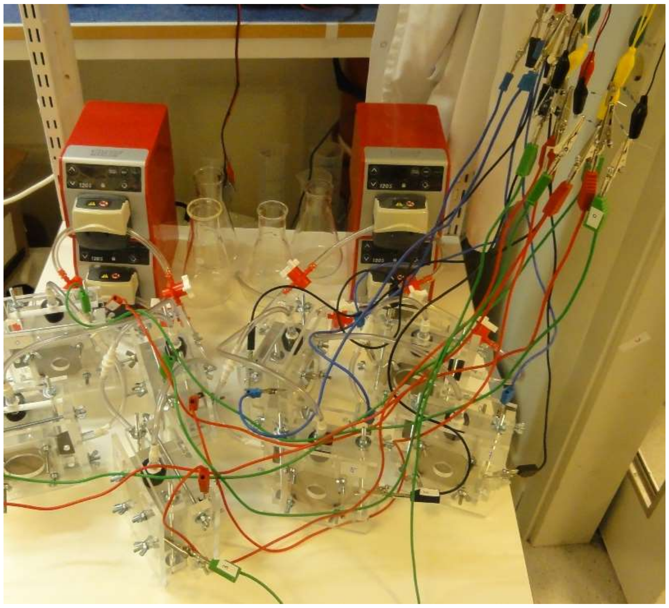

Figure S1. Schematic of a MFC and photo of the experimental setup.

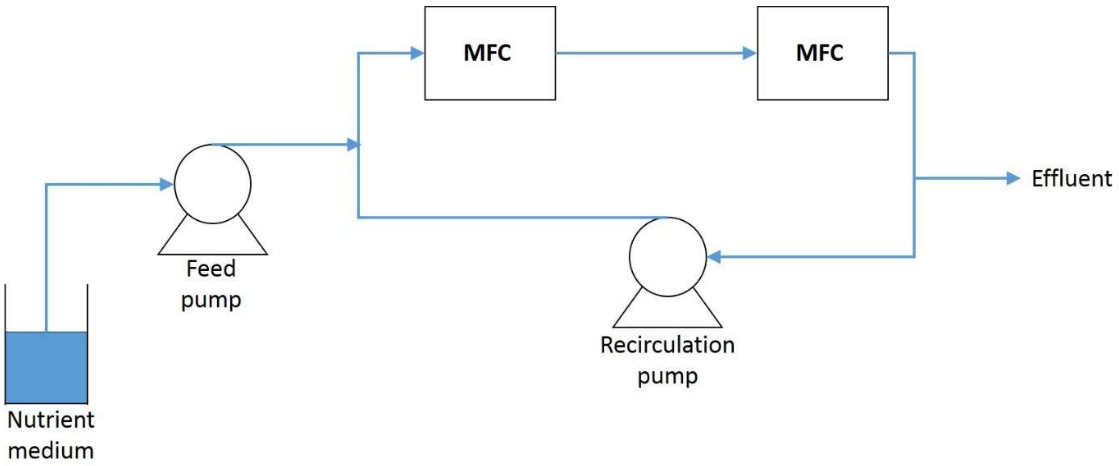

Figure S2. Schematic of a hydraulic loop containing two microbial fuel cells (MFC).

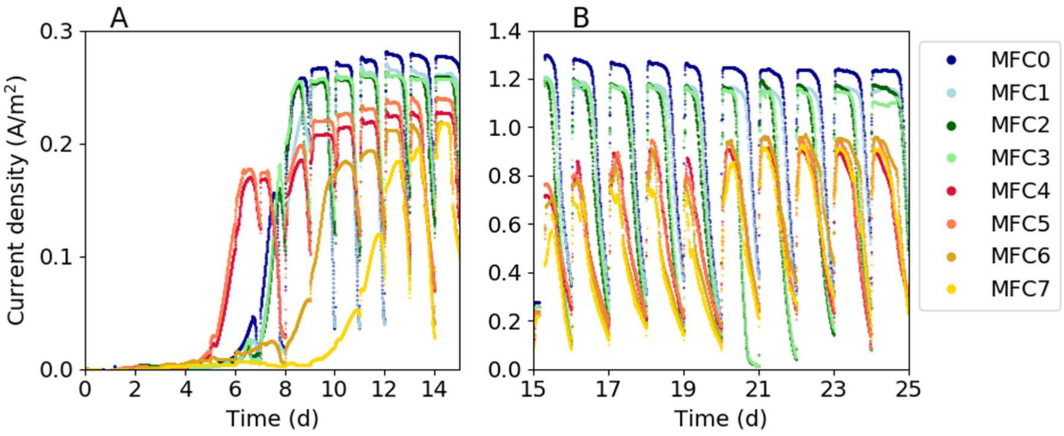

Figure S3. Electrical current density generated by the eight MFCs during (A) the initial 15 days with 1000 ohm resistors, and (B) day 15-25 with 100 ohm resistors.

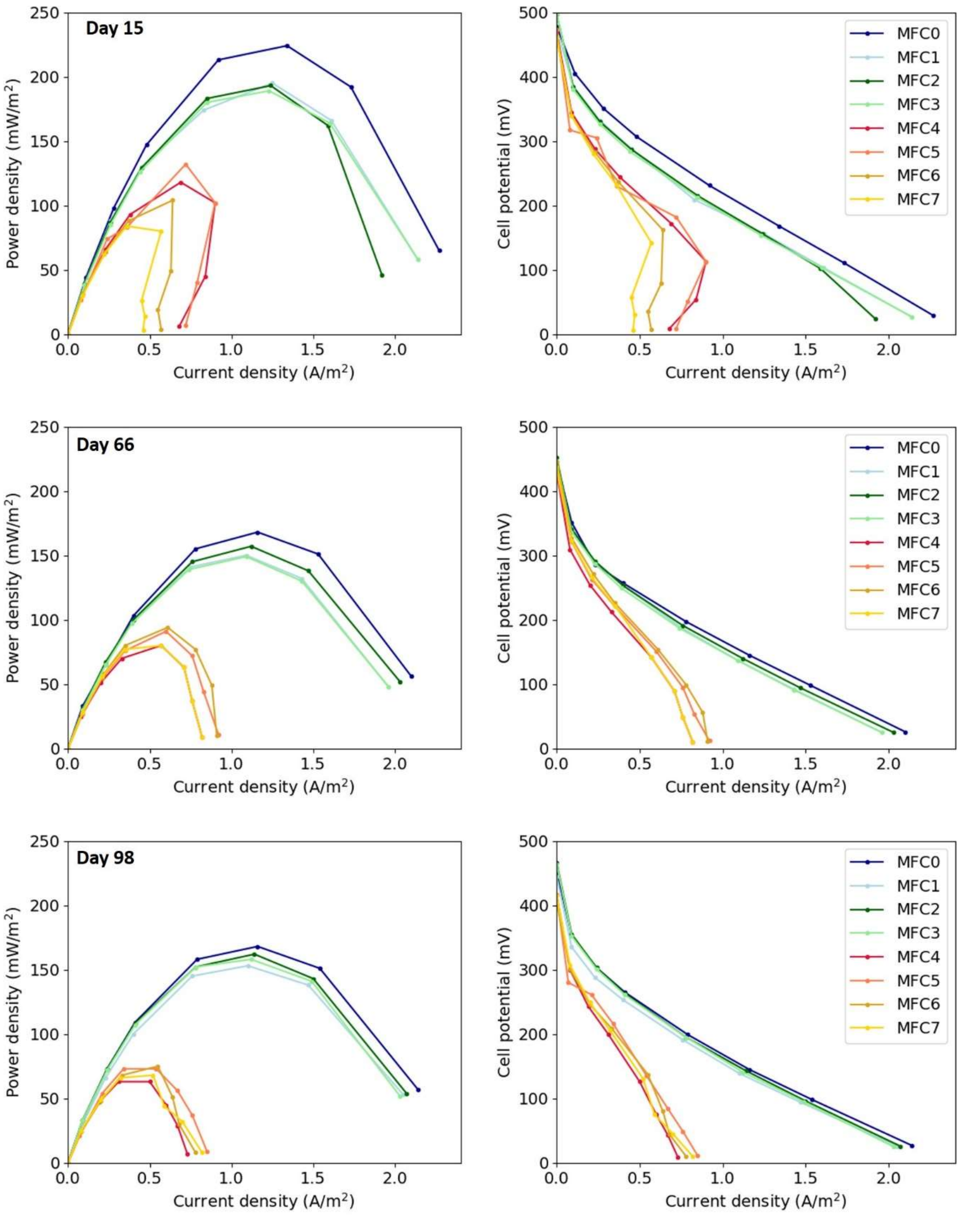

Figure S4. Polarization experiments carried out during the MFC experiment.

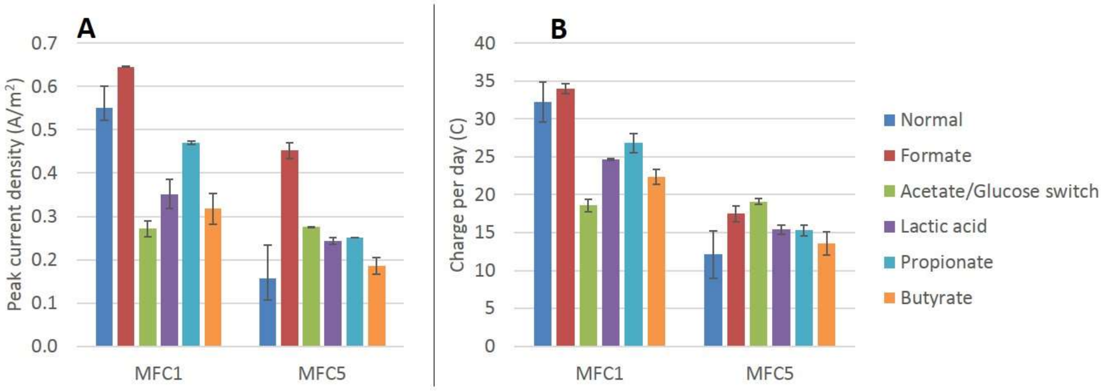

Figure S5. Peak electrical current density (A) and daily charge (B) generated by MFCs when fed with different substrates. MFC1 was enriched on acetate and MFC5 was enriched on glucose. The average of two sequential batch cycles are shown. The error bars show the minimum and maximum values.

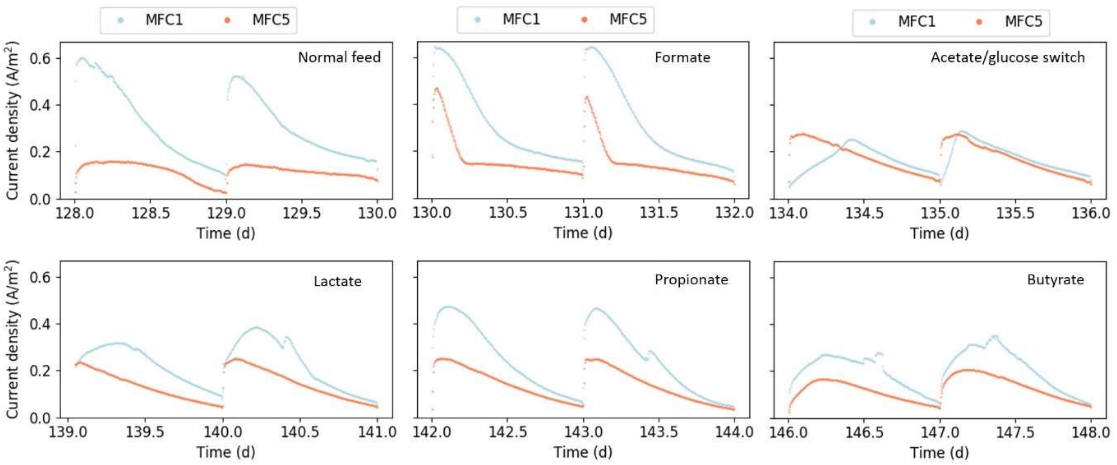

Figure S6. Current density vs time for the experiments with different substrates.

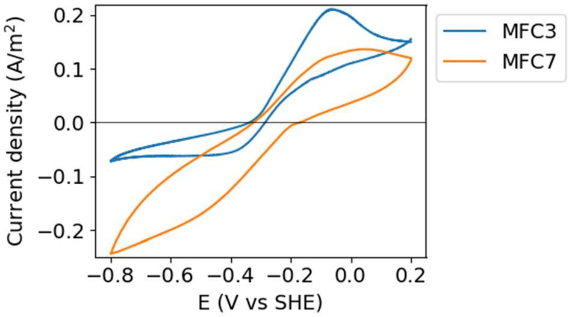

Figure S7. Results from cyclic voltammetry carried out in the end of the MFC experiment. MFC3 had been operated with acetate feed and MFC7 with glucose feed. The potential was scanned between +0.2 and -0.8 V vs SHE in three cycles at a rate of 1 mV/s. The results from the third cycle are shown. Cathodic current is negative.

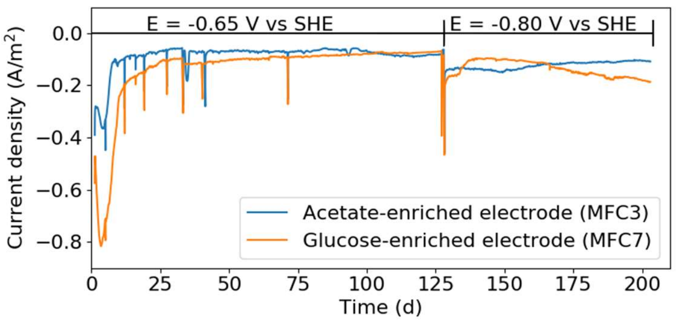

Figure S8. Prolonged enrichment of the biological electrodes from MFC3 and MFC7 under anaerobic conditions in a solution containing hydrogen ions and carbon dioxide as the only possible electron acceptors. The electrode potential was first controlled at -0.65 V vs SHE and then at -0.8 V vs SHE. Cathodic current is negative.

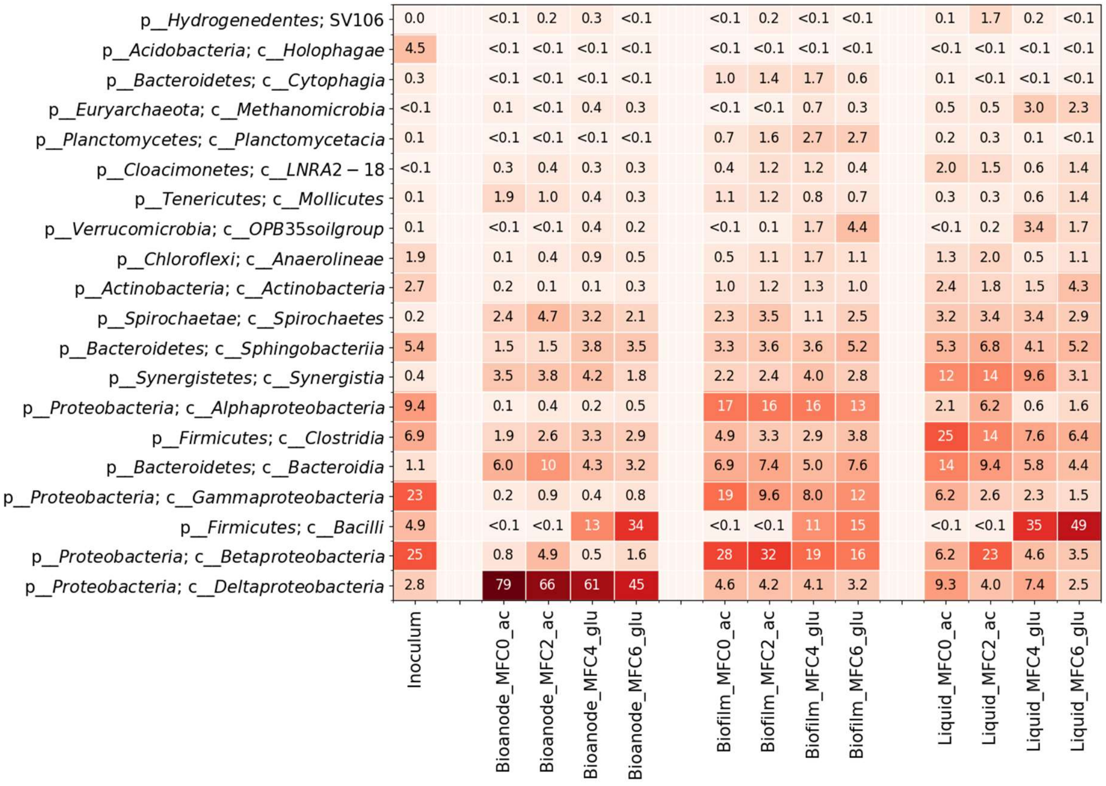

Figure S9. Heatmap showing the most abundant taxa categorized based on class. On the vertical axis, the abbreviation *c__* refers to class and *p__* refers to phylum. The labels on the horizontal axis show the samples type, the MFC, and the type of electron donor used as feed (*ac* refers to acetate and *glu* refers to glucose-feed). The numbers in the heatmap show percentage relative abundance in the sample.

Table S1. Raup-Crick dissimilarity values calculated based on 1-C_0. A value >0.95 indicates that the difference in community composition between two samples is significantly higher than could be explained by random assembly. A value < -0.95 indicates that the difference is significantly smaller than could be explained by random assembly (see Chase, et al., 2011).

	**MFC0 bioanode**	**MFC2 bioanode**	**MFC4 bioanode**	**MFC6 bioanode**
**MFC0 biofilm**	-1.00			
**MFC2 biofilm**		-1.00		
**MFC4 biofilm**			0.94	
**MFC6 biofilm**				0.06
**MFC0 liquid**	-1.00			
**MFC2 liquid**		-1.00		
**MFC4 liquid**			-1.00	
**MFC6 liquid**				-1.00

Table S2. Raup-Crick dissimilarity values calculated based on 1-C_1. A value >0.95 indicates that the difference in community composition between two samples is significantly higher than could be explained by random assembly. A value < -0.95 indicates that the difference is significantly smaller than could be explained by random assembly (see Chase, et al., 2011).

	**MFC0 bioanode**	**MFC2 bioanode**	**MFC4 bioanode**	**MFC6 bioanode**
**MFC0 biofilm**	1.00			
**MFC2 biofilm**		1.00		
**MFC4 biofilm**			1.00	
**MFC6 biofilm**				1.00
**MFC0 liquid**	1.00			
**MFC2 liquid**		1.00		
**MFC4 liquid**			0.70	
**MFC6 liquid**				0.59

Appendix S1: Description of the gas-diffusion cathodes

The base layer of the cathode was a carbon paper (AvCarb P75T with 30% wet proofing, Fuelcellearth.com). The air-facing side was painted with a mixture 20 µL/cm^2 water, 6 mg/cm^2 graphite powder (200 mesh, Alfa Aesar), and 12 mg/cm^2 PTFE. The liquid-facing side was painted with a mixture of 30 µL/cm^2 propanol, 0.15 mg/cm^2 carbon black (Cabot Black Pearls 2000), and 0.05 mg/cm^2 PTFE. The painted electrodes were sintered at 350°C for 20 min. A stainless-steel wire mesh pressed against the air-facing side of the gas-diffusion electrode served as current collector. A glass fiber filter (Munktell) pressed against the liquid-side of the cathode served as a separator between the cathode catalyst layer and the bulk liquid.

Appendix S2: Microbial community analysis methods

Wet lab procedures

DNA was extracted from samples using the FastPrep for soil DNA extraction kit. For the anode, about 2 cm^2 of carbon cloth was cut into 4 pieces and put into the extraction tube. For the cathode, which contained more biomass, about 1 cm^2 cut into four pieces was used for each sample. Samples from the inoculum and bulk liquid were centrifuged and a portion of the biomass pellet was added to the extraction tubes.

The bioanode and biofilm samples taken from MFC2 and MFC6 were divided into six different 2 cm^2 regions. DNA was extracted and sequenced from each separate region. We call the six samples from the same electrode or biofilm for extraction replicates.

The extracted DNA concentration was normalized to 20 ng/µL. PCR amplification of the V4 region of the 16S rRNA gene was carried out with the primer pair 515'F (GTGBCAGCMGCCGCGGTAA) and 806'R (GGACTACHVGGGTWTCTAAT) (Caporaso, et al., 2011, Hugerth, et al., 2014). In PCR tubes, 17 µL Accuprime Pfx supermix and 1 µL each of the forward and reverse primers (10 µM), and the DNA template were mixed. PCR reactions were done in duplicates, which were then pooled. The PCR program consisted of activation (95°C, 5 min); 30 cycles of denaturation (95°C, 20 sec), annealing (50°C, 20 sec) and elongation (68°C, 60 sec), followed by a final elongation (68°C, 10 min). The products from all samples were purified, normalized to equal concentration and pooled prior to sequencing on an Illumina MiSeq using Reagent Kit V3.

Bioinformatics

Raw sequence reads were processed in Usearch (version 10). Paired-end reads were merged with the fastq_mergepairs command allowing a maximum of 50 mismatches or at least 80% identities in the alignment. The merged reads were quality filtered using a maximum expected error (maxee) cutoff of 1 and a sequence length between 200 bp and 270 bp. The quality filtered reads were used as input to the Unoise algorithm to generate sequence variants (Edgar, 2016). A minimum abundance threshold of 4 was specified in the unoise3 command. This means that sequence variants were discarded if they were represented by fewer than 4 quality filtered reads across all samples. A count table was constructed using the otutab command by mapping the merged reads to the sequence variants with a nucleotide identity threshold of 99% required for a match. Taxonomic classification was done with the sintax algorithm (Edgar, 2016) using the Midas database (v. 213) (McIlroy, et al., 2015). The sequences in the Midas database were first trimmed to the V4 region by local alignment to the primer pair used in the study.

References

Caporaso, J.G., Lauber, C.L., Walters, W.A., Berg-Lyons, D., Lozupone, C.A., Turnbaugh, P.J., et al. (2011) Global patterns of 16S rRNA diversity at a depth of millions of sequences per sample, *P Natl Acad Sci USA* 108: 4516-4522.

Chase, J.M., Kraft, N.J.B., Smith, K.G., Vellend, M., and Inouye, B.D. (2011) Using null models to disentangle variation in community dissimilarity from variation in α-diversity, *Ecosphere* 2: 24.

Edgar, R.C. (2016) SINTAX, a simple non-Bayesian taxonomy classifier for 16S and ITS sequences, *bioRxiv* http://dx.doi.org/10.1101/074161.

Edgar, R.C. (2016) UNOISE2: Improved error-correction for Illumina 16S and ITS amplicon reads, *bioRxiv* https://doi.org/10.1101/081257.

Hugerth, L.W., Wefer, H.A., Lundin, S., Jakobsson, H.E., Lindberg, M., Rodin, S., et al. (2014) DegePrime, a program for degenerate primer design for broad-taxonomic-range PCR in microbial ecology studies, *ISME J* 5: 1571-1579.

McIlroy, S.J., Saunders, A.M., Albertsen, M., Nierychlo, M., McIlroy, B., Hansen, A.A., et al. (2015) MiDAS: the field guide to the microbes of activated sludge, *Database* https://doi.org/10.1093/database/bav062.
